# Presence of a giant mass in the interventricular septum with long follow-up

**DOI:** 10.1007/s10554-020-01893-7

**Published:** 2020-05-21

**Authors:** Jesse R. Kimman, Marc C. J. M. Kock, Marcel J. M. Kofflard

**Affiliations:** 1 Albert Schweitzer Hospital, Dordrecht, The Netherlands; 2 Erasmus University Medical Center, Rotterdam, The Netherlands

A 44-year old male patient was seen at the emergency department with dyspnea. The electrocardiogram (ECG) showed diffuse repolarization disorders (Fig. 1a). A computed tomography (CT) scan excluded pulmonary embolism but demonstrated a large hypodense mass (4.6 × 2.5 × 4.4 cm) with a density around − 90 Hounsfield Units, localized in the apical interventricular septum (Fig. 1b). The tumor was slightly inhomogeneous, protruded in the left and right ventricular cavity (straight arrows Fig. 1b), and revealed crossing septal arteries within (curved arrow Fig. 1b).

**Fig. 1:**
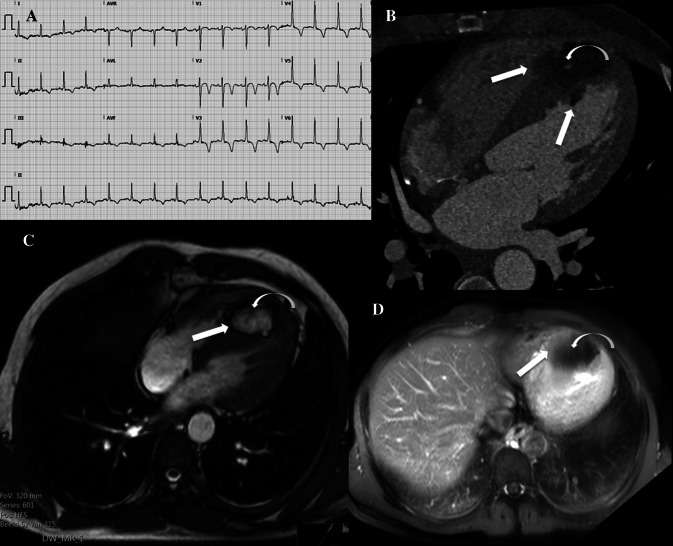
**a** Electrocardiography (ECG) showing diffuse repolarisation disorders probably due to strain. **b** CT angiography shows a large lipoid mass at the apical interventricular septum which protrudes into the left and right ventricle (straight arrows) and with a septal artery running through the mass (curved arrow). **c** Cardiac MR bSSFP image shows a chemical shift artifact (straight arrow) around the mass indicating lipoid origin; myocardial fibres are running through the mass (curved arrow). **d** Postgadolinium cardiac MR T2 weighted image with fat suppression shows lack of enhancement of the mass (curved arrow); enhancing myocardial fibres are visible as a subtle haze at the edges of the mass (straight arrow)

On magnetic resonance imaging (MRI), T1 and T2 imaging showed hyper intensity and balanced Steady-State Free Precession (bSSFP) sequence demonstrated chemical shift artefacts around the mass (straight arrow Fig. 1c), indicating a lipoid origin. Myocardial fibers were visible inside the tumor (curved arrow Fig. 1c). Post gadolinium T2 weighted images with fat suppression showed lack of enhancement of the mass (curved arrow Fig. 1d)*.* Enhancing myocardial fibres are visible as a subtle haze at the edges of the mass (straight arrow Fig. 1d). A positron emission tomography CT scan demonstrated no fluorodeoxyglucose avidity and CT angiography excluded coronary artery disease.

During admission, the patient stated that he had undergone a myocardial biopsy and thoracic CT scanning after a health check during his military duty 25 years before. Medical records were reclaimed and comparable findings were found: a 4 × 3 × 5 cm tumor suspicious for being fatty tissue. Unfortunately, the endomyocardial biopsy did not contain representative tissue.

Given the results of these tests, the tumor was considered to be a benign lipomatous mass. In contrast to cardiac lipoma, which are rounded homogeneous encapsulated masses, this non capsulated mass showed infiltration, matching the diagnosis of *lipomatous hypertrophy of the interventricular septum*. Lipomatous hypertrophy is generally seen in the interatrial septum, also known as LHIS [1]. The presence of such abundant lipomatous hypertrophy at this location is very rare and no other cases with this long time of follow-up were descripted so far.

## Electronic supplementary material

Below is the link to the electronic supplementary material.

Supplementary video (MP4 1189 kb)
